# An efficient method for chitin production from crab shells by a natural deep eutectic solvent

**DOI:** 10.1007/s42995-022-00129-y

**Published:** 2022-04-19

**Authors:** Wen-Can Huang, Dandan Zhao, Changhu Xue, Xiangzhao Mao

**Affiliations:** 1 College of Food Science and Engineering, Ocean University of China, Qingdao, 266003 China; 2 Laboratory for Marine Drugs and Bioproducts of Qingdao, Pilot National Laboratory for Marine Science and Technology (Qingdao), Qingdao, 266200 China

**Keywords:** Chitin, Food wastes, Natural deep eutectic solvents, Crab shells, Polysaccharides

## Abstract

Crab shells are an important feedstock for chitin production. However, their highly compact structure significantly limits their use for the production of chitin under mild conditions. Here, a green and efficient approach using a natural deep eutectic solvent (NADES) to produce chitin from crab shells was developed. Its effectiveness in isolating chitin was investigated. The results showed that most proteins and minerals were removed from crab shells and the relative crystallinity of the isolated chitin reached 76%. The quality of the obtained chitin was comparable to chitin isolated by the acid–alkali method. This is the first report on a green method for efficient chitin production from crab shells. This study is expected to open new avenues for green and efficient production of chitin from crab shells.

## Introduction

The global shellfishery industry generates millions of metric tons of waste every year (Yan and Chen 2015). Crustacean shells are a primary source of this waste, causing serious environmental problems and a significant waste of resources. Crustacean shells contain a useful chemical—chitin (Chen et al. 2021). Chitin has received significant attention due to its beneficial characteristics including biocompatibility, renewability, biodegradability, sustainability, bioactivity, and non-toxicity (Duan et al. 2015; Hong et al. 2018; Zhang and Rolandi 2017). It can be applied in many industries, including biomedicine, agriculture, water treatment, and cosmetics (Krajewska 2004; Zhang and Rolandi 2017).

In crustacean shells, the chitin nanofibrils are complex, containing proteins to form long chitin-protein fibers embedded in the mineral matrix (Raabe et al. 2005). Compared to shrimp shell structure, the structure of a crab shell is even more complex and compact because of its higher mineral content. Thus, it is extremely difficult to use biological methods, such as enzymatic reactions and microbial fermentation, to dissociate the chitin-protein-mineral complex to produce chitin from crab shells. Moreover, the conventional acid–alkali approach for chitin production is considered harmful to the environment. Hence, a green method for isolating chitin from crab shells is urgently needed.

Natural deep eutectic solvents (NADESs) are being increasingly considered as green and sustainable solvents. NADESs consist of natural compounds, especially primary metabolites including organic acids, sugars, and amino acids (Dai et al. 2013; Guo et al. 2019; Paiva et al. 2014). NADESs are distinguished from conventional solvents due to their favorable properties including biocompatibility, sustainability, biodegradability, non-volatility, low cost, and simple preparation methods (Xin et al. 2017).

Here, we developed a chitin isolation method using a NADES consisting of choline chloride and malic acid, to separate chitin from crab shells. In our previous work, we developed a NADES-based approach to isolate chitin from shrimp shells (Huang et al. 2018). To the best of our knowledge, this is the first report on a green chemical approach for chitin production from crab shells. The isolated chitin was characterized and the reusability of the NADES was evaluated.

## Results and discussion

### Preparation of the NADES

The NADES used in this study was composed of choline chloride and malic acid, associated by hydrogen bonding to form a eutectic mixture.

### Chitin isolation from crab shells

**Fig. 1:**
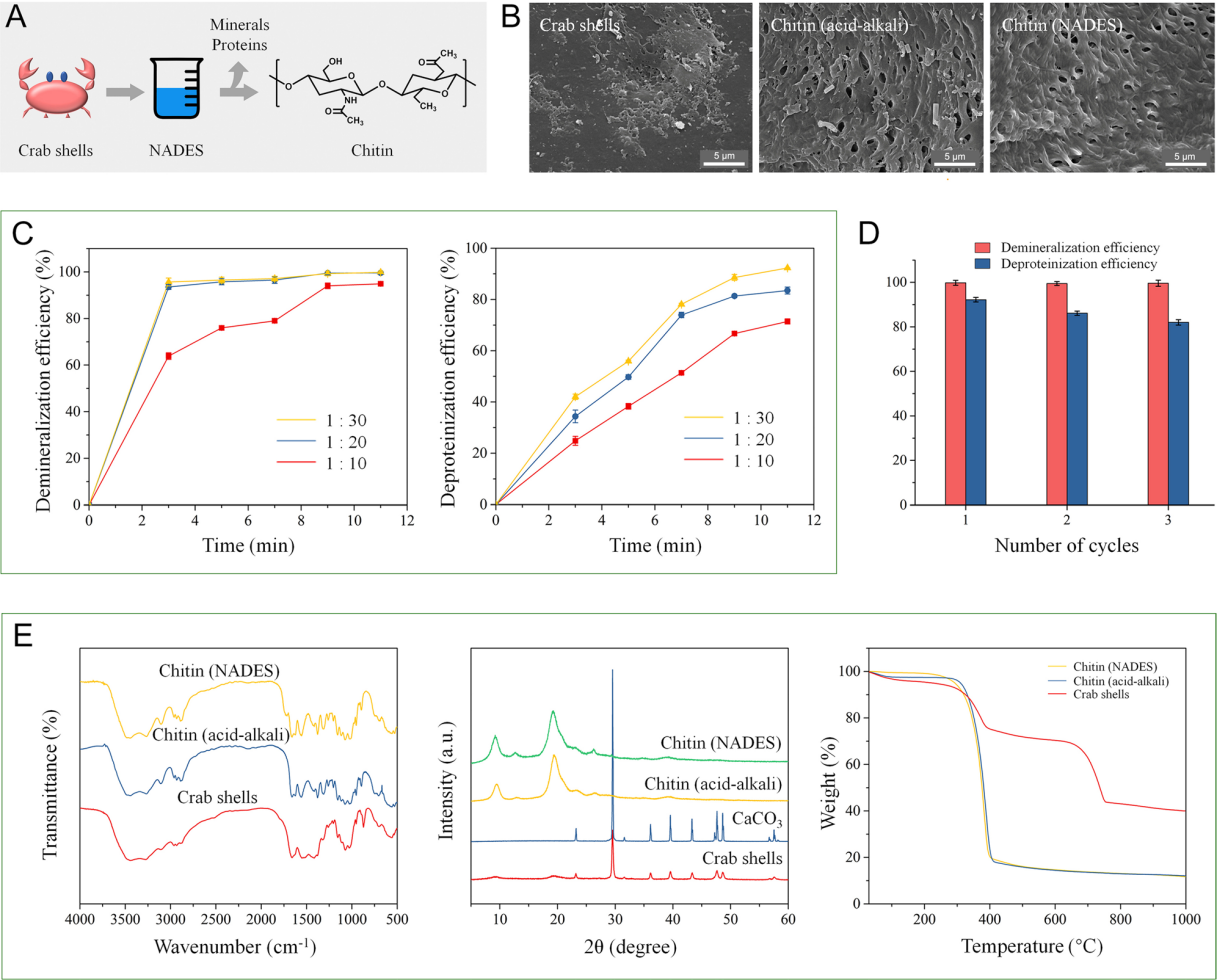
**A** Schematic diagram of the chitin isolation from crab shells by the NADES. **B** SEM images of the crab shells, chitin isolated using the acid–alkali, and chitin isolated using the NADES. **C** Demineralization and deproteinization efficiency at crab shell/NADES ratios of 1:10, 1:20, and 1:30. **D** Reusability of the NADES. **E** FT-IR spectra of the crab shells, chitin isolated by acid–alkali, and chitin isolated by the NADES; XRD curves of the crab shells, CaCO_3_, chitin isolated by acid–alkali, and chitin isolated by the NADES; TG curves of the crab shells, chitin isolated by acid–alkali, and chitin isolated by the NADES

A schematic diagram of the NADES-based chitin isolation is presented in Fig. 1A. Chitin isolation by the NADES was evaluated with various crab shell/NADES ratios (1:10, 1:20, and 1:30) and microwave irradiation times (3, 5, 7, 9, and 11 min). As seen in Fig. 1C, the demineralization and deproteinization efficiency increased with the crab shell/NADES ratio from 1:10 to 1:30. Time for microwave irradiation is another factor that can influence the chitin isolation. The demineralization and deproteinization efficiency increased when the irradiation time was increased. This result indicates that a larger amount of NADES and a longer irradiation time are beneficial for the removal of minerals and proteins from crab shells. The maximal demineralization and deproteinization efficiency reached 99.8% and 92.3%, respectively, at the crab shell/NADES ratio of 1:30 after 11 min of microwave irradiation.

### Scanning electron microscopy

The morphologies of the isolated chitin obtained by the NADES and acid–alkali, and the raw crab shells were observed by SEM. As seen in Fig. 1B, remarkable morphological changes were observed between the isolated chitin and raw crab shell. The morphologies of the chitin isolated by the NADES and acid–alkali were similar. The surface of the raw crab shells was rough with pores observed. In contrast, in the chitin isolated by NADES treatment, pores were clearly observed among the microfibrils due to the absence of proteins and minerals.

### Fourier transform infrared spectroscopy

Fourier transform infrared (FT-IR) spectra of the crab shells, and the chitin extracted using the NADES and acid–alkali are presented in Fig. 1E. The peaks of isolated chitin at 3447 cm^−1^ and 3268 cm^−1^ can be attributed to symmetric stretching vibration of NH_2_ and OH groups, respectively (Zhu et al. 2017). The amide I band split at 1660 cm^−1^ and 1626 cm^−1^ can be assigned to the existence of the intermolecular (–CO··HN–) and the intramolecular hydrogen bond (–CO··HOCH_2_–) (Rinaudo 2006). The splitting of the amide-I band was not observed in the crab shells because the amide peaks of protein overlapped with the chitin amide-I bands, indicating isolated chitin was free from protein. These absorbance peaks are the typical feature of α-chitin. No apparent differences were observed between the spectra of the chitin obtained from the NADES and acid–alkali method, suggesting chitin isolated by the NADES and acid–alkali possessed the same chemical structure as the chitin isolated by acid–alkali.

### X-ray diffraction

X-ray diffraction (XRD) was used to investigate the crystal structure of the crab shells, calcium carbonate, and isolated chitin. As shown in Fig. 1E, the chitin isolated by the NADES and acid–alkali presented diffraction peaks at approximately at 9.20°, 12.76°, 19.29°, and 26.26°, which confirmed the crystal type of the α-chitin (Zhu et al. 2017). This result indicated that the crystal structure of chitin was not changed by the NADES treatment. The diffraction peak of calcium carbonate at 29.55° was not shown in the samples isolated by the NADES and acid–alkali, indicating NADES can remove calcium carbonate. The XRD pattern of the chitin isolated by the NADES was similar to that of the chitin isolated by the acid–alkali method, suggesting complete removal of calcium carbonate. The CrI was calculated using Segal’s method. The CrI of the chitin isolated by the NADES was 75.55%, while that of the crab shells was 33.13%. The increase of CrI indicated that the NADES-based method can effectively remove minerals and proteins from crab shells.

### Thermogravimetric analysis

The results of thermogravimetric analysis (TGA) of crab shells before and after treatment are presented in Fig. 1E. The initial decomposition temperature range of raw crab shells was approximately from 150 to 300 °C, which can be assigned to the presence of proteins. Compared with the crab shells, the chitin extracted by the NADES did not show mass loss between 150 and 330 °C, suggesting proteins were not present along with chitin after treatment with NADES. The absence of mass loss between 600 and 750 °C indicates the isolated chitin was free from calcium carbonate. Additionally, the thermal stability of the chitin isolated by the NADES was similar to that of chitin obtained by acid–alkali.

### Reuse of the NADES

When the NADES, consisting of choline chloride and malic acid, is used in chitin isolation, some malic acid is consumed in removal of calcium carbonate. Hence, after each cycle, a certain amount of malic acid needs to be added to maintain the ratio of choline chloride/malic acid. As present in Fig. 1D, the NADES could be used three times without a remarkable decrease in the demineralization and deproteinization efficiency. After three times, the NADES was too viscous to be further used because proteins isolated from crab shells increased the viscosity of the NADES.

### Mechanism

Chitin isolation from crab shells using a green approach is extremely difficult because crab shells have a much higher mineral content than other crustacean shells, such as shrimp shells. Because the minerals in crab shells are mostly in the form of crystalline calcium carbonate, when the NADES was applied to the crab shells, minerals were removed by malic acid, leaving chitin and proteins. In crab exoskeletons, the minerals are deposited within the chitin–protein matrix and form a well-defined hierarchical organization (Chen et al. 2008). Thus, the strong internal structure of crab shells was weakened after the removal of minerals. NADESs are capable of breaking hydrogen bonds and have a very high ionic strength (Sharma et al. 2013). The strong hydrogen-bond network between chitin and proteins was weakened due to competing hydrogen bond formed between the chloride ions of the NADES and the hydroxyl groups, and the proteins were removed by dissolution because of the hydrogen-bond interaction with the NADES (Li et al. 2013; Sharma et al. 2013). As a result, chitin was isolated from the crab shells.

## Materials and methods

### Materials

Choline chloride was purchased from Yuanye Bio-Technology Co., Ltd. Malic acid, HCl, and NaOH were purchased from Sinopharm Chemical Reagent Co., Ltd.

### Preparation of the NADES

Choline chloride and malic acids were mixed with a molar ratio of 1:1 at 80 °C until formation of a homogeneous liquid.

### Isolation of chitin from crab shells

Chitin isolation from crab shells by the NADES was performed as follows. The ground crab shells and NADES were mixed at various shell/NADES ratios (1:10, 1:20, and 1:30). Next, the shell/NADES mixture was heated by microwave irradiation at 700 W. The isolated chitin was then separated from the mixture by centrifugation. The sample was then rinsed with distilled water followed by drying in a vacuum oven.

For comparison with the NADES-based chitin isolation, acid–alkali-based chitin isolation from crab shells was conducted. Demineralization was performed by treating the crab shells with a 5% (w/v) HCl solution with a HCl solution-to-shell ratio of 10 ml/g at room temperature for 1 h. Next, the demineralized sample was collected by centrifugation followed by deproteinization with 10% (w/v) NaOH with a NaOH solution-to-shell ratio of 10 ml/g at 95 °C for 1 h. The isolated chitin was then rinsed with distilled water followed by drying in a vacuum oven.

The mineral content was measured by heating the samples (1–2 g) at 525 °C to a constant weight in a muffle furnace. The demineralization efficiency was calculated using the following equation:

$$\mathrm{Demineralization }\left(\mathrm{\%}\right)=\frac{{M}_{0}-M}{{M}_{0}}\times 100\%$$

where *M*_0_ and *M* are the mineral contents of the crab shells and isolated chitin, respectively.

The protein content was determined by the Bradford method (Bradford 1976). Briefly, Coomassie Brilliant Blue G-250 (100 mg) was dissolved in ethanol (95%, 50 ml). Then, phosphoric acid (85%, 100 ml) was added, and the total volume of the solution was adjusted to 1 L with distilled water. Next, a 0.5 g sample was added to 5% NaOH, and the mixture was stirred at 95 °C for 1 h, followed by filtering and dilution. Then, the resulting sample was added to the solution, and the absorbance was measured at 595 nm after 2 min. The deproteinization efficiency was calculated using the following equation:

$$\mathrm{Deproteinization }\left(\mathrm{\%}\right)=\frac{{P}_{0}-P}{{P}_{0}}\times 100\%$$

where *P*_0_ and *P* are the protein contents of the crab shells and isolated chitin, respectively.

To evaluate reusability, the NADES was recycled three times without purification after chitin isolation.

### Characterization

The surface morphologies of the crab shells and isolated chitin were observed by SEM (JEOL JSM-840) with an acceleration voltage of 20 kV. Before observation, all the samples were coated with platinum by vacuum sputtering. The FT-IR spectra were recorded on a FT-IR spectrometry (Thermo Scientific Nicolet iS10) over the frequency range of 4000–500 cm^−1^ with a resolution of 4 cm^−1^. The XRD patterns were recorded on an X-ray diffractometer (Rigaku Miniflex 600) using Cu Kα radiation at 40 kV. The diffraction data were collected at a scanning rate of 5° min^−1^ from 2θ = 5 − 60°. The relative crystallinity index (CrI) was calculated by the Segal method:

$$\mathrm{CrI }\left(\mathrm{\%}\right)= \frac{{I}_{110}-{I}_{\mathrm{am}}}{{I}_{110}} \times 100\%$$

where *I*_110_ is the peak intensity of the diffraction at 2*θ* ≈ 20°, which represents both the crystalline and amorphous region material, and *I*_am_ is the diffraction intensity of the amorphous fraction at 2*θ* ≈ 18°. TGA was performed under a nitrogen atmosphere at a heating rate of 10 °C min^−1^ by a thermogravimetric analyzer (NETZSCH TG 209 F3).

## Conclusions

In the present study, we developed a NADES-based method for chitin production from crab shells. The experimental results indicated that most protein and minerals were removed, and the quality of the isolated chitin was superior to that isolated by other methods. In addition, the NADES can be reused three times without purification. This method was proved to be green, efficient, facile, and sustainable. The chitin produced by the proposed method was comparable to the chitin isolated by the acid–alkali method. This study provides a method for the sustainable production of chitin from crab shells.

## Data Availability

The datasets generated during and/or analysed during the current study are available from the corresponding author on reasonable request.
